# 

**Published:** 1918-02-16

**Authors:** 


### CONTENTS.

PAGE

**Having It Out** ... ... ... ... ... ... 407

**The Physical Fitness of the Nation** ... ... 408

**Hospital and Institutional News** ... ... ... 409

Munificent Bequests by a Hospital Chairman—Mr. Coles' Successor at Cambridge—The Staff Record at Halifax Royal Infirmary—Hospital Buildings after the War—Doctors' Fees for Army Patients—The Hospital Standard in Leeds—The First Two 1917 Hospital Reports—Surrender Week—A Suggestion to Hospital Collectors—Municipal Consultations—Discharged Epileptic Soldiers—The Value of Medical Expert Evidence—Dental Treatment for Mothers—Baby Care in Plymouth—Illegitimacy and Death—Poisonous Trees—The Status of Chemists—Reflections on Peace—This Week's Drug Market.

**Glimpses Backward** ... ... ... ... ... 413

III. The Medieval Attitude to Science.

**Lord Lister** ... ... ... ... ... ... ... 415

IV. Surgery in Pre-Listerian Days.

**Gifts and Bequests** ... ... ... ... ... ... 416

**Reconstruction and the Poor Law** ... ... 417

The Unification of the Public Health Services.

**Passing Events and Topics** ... ... ... ... 418

**The London Hospital's Quinquennial Appeal** 419

By Viscount Knutsford, Chairman.

A Million Half-crowns—Viscount Knutsford's Appeal.

**National Insurance Act Developments...** ... 420

The Lords' Amendments to the New Bill.

**The College of Nursing (Limited by Guarantee)** 422

List No. 4 of Training-schools of Registered Nurses.

PAGE

**The Matrons' and Sisters' Department** ... 421

Round the Hospitals.

Duchess of Bedford Helps British Nurses—Miss E. Musson's Excellent Speech—Newcastle's Wounded Friends' Travelling Fund—Sir Arthur Stanley Misrepresented—Liverpool Favours Nurses' Unity—Sisters' Allowances at St. Bartholomew's.

**The Status of Nurses...** ... ... ... ... ... 423

Some Unfounded Statements at Liverpool.

Sir Arthur Stanley's Letter: The Status of Nurses.

**State Registration** ... ... ... ... ... ... 424

R.B.N.A. Speakers' Hostility to the College: Lay Governors and Hospital Chairmen Attacked.

**Institutional Needs** ... ... ... ... ... ... 427

**A Complete System of Nursing** ... ... ... 428

Reviewed by a Physician and a Trained Nurse.

The Physician's View—What the Trained Nurse Thinks.

**Matrons' and Sisters' Appointments** ... ... iv

**The Editor's Letter-Box** ... ... ... ... iv

Women as Sanatoria Superintendents.

**The Institutional Worker** .. (Suppl.) 1

Extending a Hospital Engineer's Work: Answer to January Question.

Crockery Washing in a Nurses' Home: Answer to January Question.

February Questions.

Questions Invited.

Enquiries and Answers.

War Matters.

Employment and Training.

The Sick and in Need.

The Housekeepers' Department.

Miscellaneous.

